# Predicting the Reasons of Customer Complaints: A First Step Toward Anticipating Quality Issues of In Vitro Diagnostics Assays with Machine Learning

**DOI:** 10.2196/medinform.9960

**Published:** 2018-05-15

**Authors:** Stephane Aris-Brosou, James Kim, Li Li, Hui Liu

**Affiliations:** ^1^ Department of Biology University Of Ottawa Ottawa, ON Canada; ^2^ Ortho Clinical Diagnostics Raritan, NJ United States

**Keywords:** post market surveillance, QC chemistry results, complaint data, CART, adaptive boosting

## Abstract

**Background:**

Vendors in the health care industry produce diagnostic systems that, through a secured connection, allow them to monitor performance almost in real time. However, challenges exist in analyzing and interpreting large volumes of noisy quality control (QC) data. As a result, some QC shifts may not be detected early enough by the vendor, but lead a customer to complain.

**Objective:**

The aim of this study was to hypothesize that a more proactive response could be designed by utilizing the collected QC data more efficiently. Our aim is therefore to help prevent customer complaints by predicting them based on the QC data collected by in vitro diagnostic systems.

**Methods:**

QC data from five select in vitro diagnostic assays were combined with the corresponding database of customer complaints over a period of 90 days. A subset of these data over the last 45 days was also analyzed to assess how the length of the training period affects predictions. We defined a set of features used to train two classifiers, one based on decision trees and the other based on adaptive boosting, and assessed model performance by cross-validation.

**Results:**

The cross-validations showed classification error rates close to zero for some assays with adaptive boosting when predicting the potential cause of customer complaints. Performance was improved by shortening the training period when the volume of complaints increased. Denoising filters that reduced the number of categories to predict further improved performance, as their application simplified the prediction problem.

**Conclusions:**

This novel approach to predicting customer complaints based on QC data may allow the diagnostic industry, the expected end user of our approach, to proactively identify potential product quality issues and fix these before receiving customer complaints. This represents a new step in the direction of using big data toward product quality improvement.

## Introduction

Connected and so-called smart meters and other tools have transformed virtually every industry by enabling new functions and capabilities such as continuous monitoring, control, optimization, and autonomy [1]. This is particularly true in the health care industry, which deployed analytical systems ranging from electronic health records (EHRs) to clinical decision support systems [2]. Connected systems also include in vitro diagnostic (IVD) analyzers, which work with different assays that measure a number of markers in patients’ blood samples such as sodium or potassium, as well as other biomarkers such as troponin—which altogether are called “assays.” Being connected, their manufacturers can monitor the analyzers’ output in real time through encrypted, two-way interactive connections. As such, manufacturers can potentially quickly detect issues and act promptly to resolve the problem.

However, the sheer amount of data generated by these connected systems is such that big data analytics are required [3]. For this, a number of platforms have been developed, going from statistical tools such as R [4], to dedicated business intelligence and data mining tools. These platforms can then generate queries, reports, and perform online analytics processing, as well as data mining [2]. These aggregated data can then be used to perform one of three kinds of analytics: (1) descriptive analytics that permit the visualization of the data; (2) predictive analytics that try and predict the future of a system from its past behavior; and (3) prescriptive analytics that make recommendations about the best way to resolve a particular issue [5]. However, different health analytics contexts may require different approaches, as in the case of quality control (QC) data logged by analyzers.

As QC data are routinely used to monitor the performance of IVD and identify signals that may indicate a performance change, a number of approaches have been developed. These range from panels of experts that submit monthly reports [6], to automated systems that resort to summary statistics computed over temporal windows [7-11]. Although simple linear models can be used to monitor these complex systems [12], machine-learning algorithms have already proved capable of generating highly accurate predictions [13,14]. However, past approaches mostly have explored simple tools such as decision trees and other standard classifiers [15] and have not (1) Explored more sophisticated algorithms such as adaptive boosting [16] and (2) In the context of noisy and moderately large dataset—that are, hence, not always amenable to deep learning as recently deployed in the context of EHRs [17]. One aspect that has rarely been integrated into the analysis of QC data is its relation with customer data: when a shift in performance of a test assay is identified, what is its impact on the user (customer)? Will this trigger a complaint about QC? If the complaint is specific, such as “QC high” or “accuracy low,” can we learn something about the quality of the data from the combination of those specific complaints?

The objective here is therefore to integrate these two kinds of data, QC data and customer complaints, to be able to predict specific QC issues, while accounting for intrinsic issues pertaining to customer data. Indeed, customer complaint databases have at least three inherent limitations that need to be considered when designing a prediction tool. First, complaint databases may contain inaccurate, incomplete, untimely, or unverified information [18]. Second, incidence may be under [19,20] or overreported [21]. For instance, certain advertising or regulatory actions may result in increased reporting [22], which could ultimately result in an overwhelming important signal with noise. Third, despite the best efforts of complaint handling professionals, errors while curating complaints (eg, misclassification of complaints) occur [23]. However, it is possible that by focusing solely on errors directly related to QC, or even by binning particular errors into larger categories (eg, “QC high” and “QC shift high” in the same category), it might alleviate some of these reporting issues.

Here, based on a particular connected IVD analyzer, we show that integrating QC data with a database of customer complaints can be used to predict which type of issues customers complain about. We hypothesized that connected systems can be utilized more efficiently by utilizing the collected QC data more efficiently and more specifically by resorting to machine-learning algorithms. We show that it is possible to identify product issues more proactively, which makes it possible to act on these before they trigger a customer complaint. We further show that some filtering of the complaint data (denoising) improves the accuracy of issues prediction. This work represents a first step toward meeting the recent plan from the US Food and Drug Administration (FDA) to leverage on big data to improve device performance and health care [24].

## Methods

### Data Collection

#### e-Connectivity Data

Data were collected using the e-Connectivity application’s chemistry results, manufactured by Ortho Clinical Diagnostics (Raritan, New Jersey). This feature allows the manufacturer to pull information remotely from equipment installed at customer sites, which are themselves distributed throughout the world. The data retrieved in this study were generated by Ortho Clinical Diagnostics’ VITROS analyzers of the 5,1 FS series, the 5600, 4600, 3600, or ECi/ECiQ Systems, that all log the same kind of information through e-Connectivity. Only QC data were extracted to avoid complications linked to patients’ data (identifiability, variability, etc).

The e-Connectivity data contain information relative to the assay, serial numbers reflecting its origin, the measured concentrations, as well as some information relative to the analyzer itself (see Table 1 and Multimedia Appendix 1 for a full description of the e-Connectivity variables). We focused on five assays, here recoded as “assay A” to “assay E.” The data pulled ranged from March 16, 2016 00:00:20 EST to June 14, 2016 23:38:51 EST, a total of 90.98 days, and contained 824,885 QC logs across the five assays. To assess the effect of the training period, we constructed a second set of data limited to the last 45 days of this 90-day set.

#### Customer Data

The corresponding customer complaint data were obtained by querying the product complaint database of the same manufacturer for the same time window as the QC data. Customer data contained information with respect to the assay for which an issue is reported, the call area (error code), and other information related to the assay (see Table 1 and Multimedia Appendix 2 for a full description of the customer variables; Multimedia Appendix 3 list the call areas reported over the five assays employed here). These data contained a total of 7999 logs. Across the five assays tested here, a total of 99 call areas were found. The goal here is to predict these call areas from the QC data.

#### Records Matching

The only fields that are shared between QC and customer data are assay name, J numbers, and lot numbers (Table 1). As each analyzer has a unique J number, we used this shared information to match QC samples with customer data. Although this approach works in most cases, there are instances when the same customer processes multiple samples, potentially from multiple analyzers, but logs only one call. Thus, the data that will be used to train the predictive algorithms are, in essence, noisy.

### Predictive Classifiers

#### Feature Definitions

**Figure 1:**
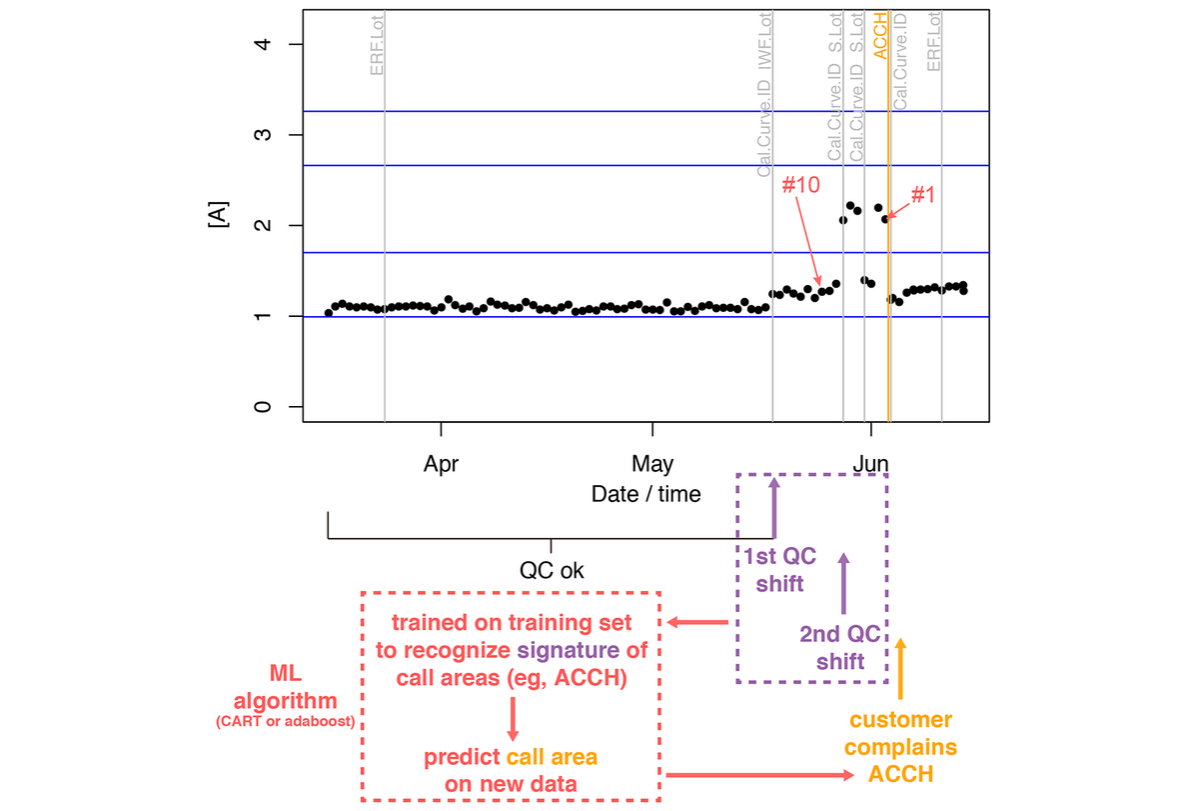
Feature definitions based on a typical sample logged in e-Connectivity. Assay concentrations (here for assay A) are plotted as a function of time. Horizontal blue lines show the modes of the density of sample means (our estimated verifiers). Vertical gray lines show timing of maintenance activities (change of calibration curves, etc). The orange vertical line shows when the customer placed a call—for “accuracy high” (ACCH; indicates the measured concentration is suspected of being higher than the actual value) in this example. The concentration reading just before this call (“#1”) and 10 e-Connectivity logs before it (“#10”) are indicated in red. Our machine-learning (ML) algorithms (in red) aim at learning the signatures (in purple) of call areas (orange) from a training set, to be able to identify those call areas, before a customer complains.

To find predictors of customer complaints based on QC data, we need to define operational variables, which are called features. These features were defined by inspecting a typical log of the system (Figure 1). From this, two types of features were defined, based on (1) concentration readings and (2) maintenance events (eg, change of calibration).

**Table 1:** List of the fields logged by e-Connectivity (that includes quality control, QC data) and by the customer complaint system. Corresponding abbreviations are shown.

Data and Abbreviation		Short description
**e-Connectivity**		
	Assay	Abbreviation of assay name (recoded here)
	J Number	Unique identifier assigned to each analyzer placed
	F Concentration	Concentration of solute (assay); QC^a^ result
	Units	Unit of measured concentration (mmol/L)
	F Concentration (SI)	Concentration of solute (assay); QC result
	Units SI	Unit of measured concentration (SI)
	Reagent Lot Number	Reagent lot number
	S Gen	Manufacturing generation number
	S Lot	Manufacturing lot number
	ERF Lot	Electrolyte reference fluid lot
	IWF Lot	Immuno-wash fluid lot number
	Control Lot Number	Performance verifier lot number
	Cal Curve ID	Calibration curve ID
	Result ID	Unique identifier (encrypted) of QC result
	Sample Name	Unique identifier (encrypted) of sample name
	Time Metering	Time stamp of concentration log through e-Connectivity
	Total Dilution	Dilution factor
	Operator Dilution	Operator requested dilution
	Body Fluid	Fluid type (serum, plasma, or urine)
**Customer**		
	Create Audit Date	Time stamp of when complaint was placed
	Call Subject	Same as assay in e-Connectivity
	Call Area	Classification of concern or problem of the product or the analyzer-generated condition
	Resolution	Term describing how the complaint was resolved
	Complaint Number	Unique identifier of each complaint
	Customer Number	Unique identifier of each customer
	J Number	Analyzer serial number
	Lot number	Reagent lot number
	Region	Geographic region where complaint was placed
	Call Status	Current call status of complaint (closed or open)
	Problem description	Free-text field describing the complaint

^a^QC: quality control.

Concentration readings departing from expected values can be thought of as the prime trigger of customer complaints. Obviously, their absolute value with no other context has no predictive value (as long as it is not outside of the biological range) for QC data, and therefore, we should focus on departure from verifiers, which are known concentration readings produced during manufacturing. However, these verifiers are not logged by e-Connectivity and are only available as PDF files, which cannot be easily parsed. Customers may also choose to use QC material that is manufactured by a third-party, which further complicates the retrieval of verifier information. As a workaround, we calculated mean concentrations by samples, estimated the density kernel of these sample means, and determined the location of all the modes (Multimedia Appendix 4). We assumed that each mode corresponds to a verifier: the closest mode to each QC reading logged in e-Connectivity was assumed to be the verifier concentration. We then used the relative differences between concentration readings and estimated verifier. On the basis of this, we defined four features according to concentration readings just before customer call (log “#1” in Figure 1) and of the average logs two, five, and ten time points before the call (orange vertical bar in Figure 1). Because variability of QC logs may also signal issues, we defined three more features by SDs of the two, five, and ten concentration logs before customer call.

Customers may notice suboptimal performance of a machine and decide to try and resolve the issue on their own and place a call for assistance only if they cannot resolve the issue. We therefore defined features based on different maintenance events logged by the system (six in total): change of S Gen, S Lot, ERF Lot, IWF Lot, Control Lot Number, and Cal Curve ID. We considered both the timing of the last event before the call and the number of such events before the call. This led us to define 12 additional features based on maintenance events, for a grand total of 19 features (Table 2). A twentieth feature was defined as the time it takes for a customer to call since the last e-Connectivity reading (at “#1” in Figure 1).

Because the use of only “positive samples” (samples that led to a customer call) to train our algorithms would bias any prediction toward overpredicting calls, we also defined features for “negative samples.” These are QC samples that did not generate any customer complaints. If *n* calls were logged for a given assay (among the 7999 logs in total), we drew *n* such negative samples. We calculated the features as above by drawing a cutoff time at random (from a uniform distribution limited by start and end time of QC logs for a given sample) that plays the role of a customer call in the positive samples. In this case, the call area (error code) is “OK”—giving then a total of 100 call areas that we want to predict.

#### Machine-Learning Algorithms

These 20 features were used as predictors during the training of machine-learning algorithms, whose goal was to classify (predict) the qualitative nature of problem represented by each call area. Two such algorithms were used here: a simple one, based on decision trees [25], and a more sophisticated one that recently proved very successful in one of our applications [26].

Decision trees represent one of the simplest type of classifier, with Classification and Regression Trees (CART) being one of the most basic algorithms. We employed the algorithm implemented in the tree library [27] version 1.0-37 in R version 3.2.3 [4]. Unlike CART, adaptive boosting relies on an iterated process that proposes boundaries in the space of predictors, each giving rise to a weak classifier; the final classifier then combines these different weak classifiers, emphasizing misclassifications, to create a final strong classifier [16]. The adabag library version 4.1 [28] was used. To avoid overfitting with both algorithms, each dataset (the 90- and the 45-day sets) was split into two subsets, where two-thirds of the data were used as a training set and the remaining one-third used to test performances (compute misclassification or error rate from the confusion table). Because of the many ways to split the data in a 2:1 ratio, we repeated this cross-validation exercise for 2500 random such 2:1 splits of the data, for both classifiers. Such a cross-validation experiment can also be seen as a means to prevent overfitting the data with a complex model.

#### Data Denoising

Over the 99 call areas employed so far, some are not directly related to QC, and those related to QC might share some characteristics. Both issues can create some noise, which can easily be filtered out of the data. We therefore created two filters, one that removes all non-QC related call areas (essentially, all error codes starting with a “Z” in Multimedia Appendix 3, as they are related to a misconfiguration of the analyzer) and one that bins some call areas. The first filter reduced the complaint data from 7999 to 572 logs and from 99 to 21 call areas by eliminating error codes unrelated to QC. The second filter, binning all QC high (QC high, QC Drift High, QC Shift High) together and all QC low (QC low, QC Drift Low, QC Shift Low) together, further reduced the number of call areas from 21 to 17. Applied both to the 90- and the 45-day data, these filtering steps led to four additional datasets. Our expectation was that these denoising steps would improve the performance of our classifiers, as reducing the number of categories from 99 to 17 simplifies the classification problem. The R code developed in this study is available from GitHub (sarisbro account); the QC data we used are proprietary, contain no patients records, but the variables used are listed in Table 1.

**Table 2:** List of the features used in the predictive modeling. Note that a “cutoff” represents the time when a customer calls in the case of “positive samples” (when there is an actual complaint), or the time drawn at random in the case of “negative samples” (see Methods).

Feature name	Definition
MostRecentConcentration	Assay concentration reading just before cutoff
TwoMostRecentConcentrationMean	Mean concentration for the two readings before cutoff
FiveMostRecentConcentrationMean	Mean concentration for the five readings before cutoff
TenMostRecentConcentrationMean	Mean concentration for the ten readings before cutoff
TwoMostRecentConcentrationSD	SD of concentration for the two readings before cutoff
FiveMostRecentConcentrationSD	SD of concentration for the five readings before cutoff
TenMostRecentConcentrationSD	SD of concentration for the ten readings before cutoff
NbPriorSGenChange	Number of S Gen changes before cutoff (since start of QC sample)
NbPriorSLotChange	Number of S Lot changes before cutoff
NbPriorERFLotChange	Number of ERF Lot changes before cutoff
NbPriorIWFLotChange	Number of IWF Lot changes before cutoff
NbPriorContLotNumChange	Number of Control Lot Number changes before cutoff
NbPriorCalCurveChange	Number of Calibration Curve changes before cutoff
TimeSinceLastSGenChange	Time elapsed since last S Gen change before cutoff
TimeSinceLastSLotChange	Time elapsed since last S Lot change before cutoff
TimeSinceLastERFLotChange	Time elapsed since last ERF Lot change before cutoff
TimeSinceLastIWFLotChange	Time elapsed since last IWF Lot change before cutoff
TimeSinceLastContLotNumChange	Time elapsed since last Control Lot Number change before cutoff
TimeSinceLastCalCurveChange	Time elapsed since last Calibration Curve change before cutoff
TimeToComplain	Time elapsed since last e-Connectivity log before cutoff

## Results

### Very Low Error Rates Even With Noisy Data

**Figure 2:**
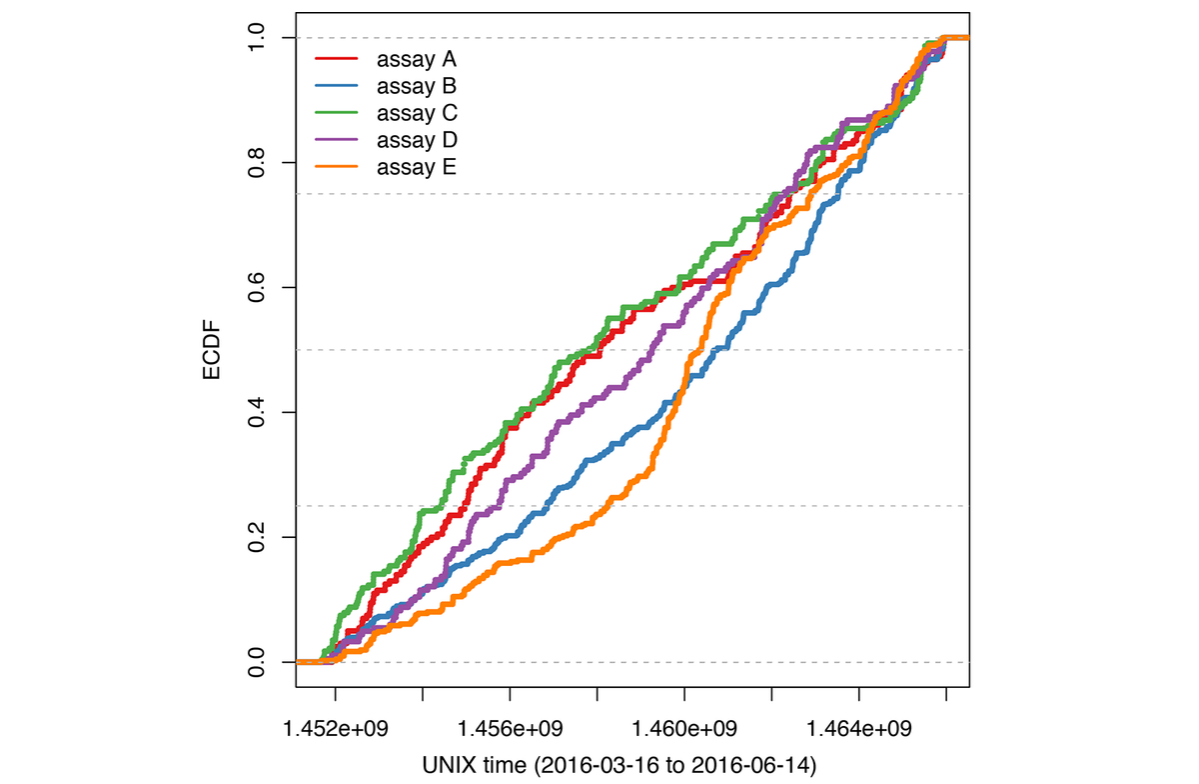
Empirical cumulative distribution function (ECDF) of customer complaints. The ECDF was plotted for the five assays considered. The horizontal gray bars represent the first, second, and third quartiles. Each assay is color-coded as shown (inset).

To predict which call areas are used when a customer complains only using QC data (Figure 1), we implemented two machine-learning algorithms that we ran on five different assays. As expected, CART showed error rates that were higher than those obtained with adaptive boosting, but both algorithms did much better than chance, with median error rates as small as 8% (Multimedia Appendix 5). Over the 90-day sample, each assay had triggered different numbers of complaints (assay A: 200, assay B: 835, assay C: 227, assay D: 182, assay E: 410, Multimedia Appendix 6), so that we expected that assays with larger number of complaints would have larger predictive power, but that was not the case (*t*_3_=1.027, *P*=.38). Instead, the temporal dynamics of customer complaints, which increased in the second half of the 90-day period (Figure 2), affected error rates (Multimedia Appendix 5): in particular, the first quartile of the empirical cumulative distribution of customer complaints was a strong predictor of the error rate (adaptive boosting: *t*_3_=4.103, *P*=.03). This suggests that it is easier to predict a call area (the type of a problem) for assays that quickly generate complaints.

### Importance of Timing and Variability in Predicting Call Type

Adaptive boosting computes a measure of importance for each feature. Multimedia Appendix 6 shows that time to complain was the most important feature across all five assays tested. The second most important features were mostly those involving the timing of maintenance events, followed by the variability of concentrations (SDs) of the QC material. Unexpectedly, the actual concentration means (last two, five, or ten) were systematically the least important features for predicting call areas.

### Misclassification Increases When Time to Complain Is Ignored

The previous results included time to complain as a feature; again, this is the time lag between the last QC reading by the system and the time when a customer placed a complaint (Figure 1). This is unrealistic, as in a real application, we would not know when a customer is going to complain. As a result, we assessed the impact of removing the time to complain feature from our classifiers. Both CART and adaptive boosting were affected by this removal, even if all five assays still had median error rates *<* 50% and as low as 20% with adaptive boosting (Multimedia Appendix 5). This increase in error rate after removal of this feature shows that time to complain is an important determinant of a complaint, which in turn suggests that customers are quick to complain after detecting a QC shift.

Note, however, that this removal of the most important feature did not affect the relative importance of the other features: those involved in the timing of maintenance events and those describing the variability of concentrations (SDs) were still the most important predictors (Multimedia Appendix 7).

### Shorter Datasets Increase Accuracy

The results above suggest that the rate of complaint may affect performance. But it is unclear if longer training periods can benefit the performance of our algorithms. To test this, we subset the 90-day data to its last 45 days. When all the features were used to train the algorithms, all classification error rates decreased (Multimedia Appendix 5). A consistent pattern was observed when time to complain was also removed from the feature list (Multimedia Appendix 5). This suggests that the statistical process underlying call areas is nonstationary in time (ie, is time-heterogeneous). This hypothesis was supported by the change in error rate of assay E, which was the worst performer with the 90-day data but became one of the best one with the 45-day data, where a sharp increase in customer complaints can be observed at the beginning of this period (Figure 2, and Multimedia Appendix 6). It is therefore possible that training periods might have to be adjusted as calls are coming in: small number of complaints may require longer training periods, whereas an increase in complaint volume may necessitate reducing the training period in real time. On the other hand, it is also clear from Multimedia Appendix 6 that by shortening the training period, the number of call types was also reduced, so that the algorithms needed to predict fewer categories, which also contributed to lowering error rates. Therefore, shorter datasets may increase accuracy, but at the cost of being less general in the type of calls that can be predicted.

### Data Denoising Increases Accuracy

**Figure 3:**
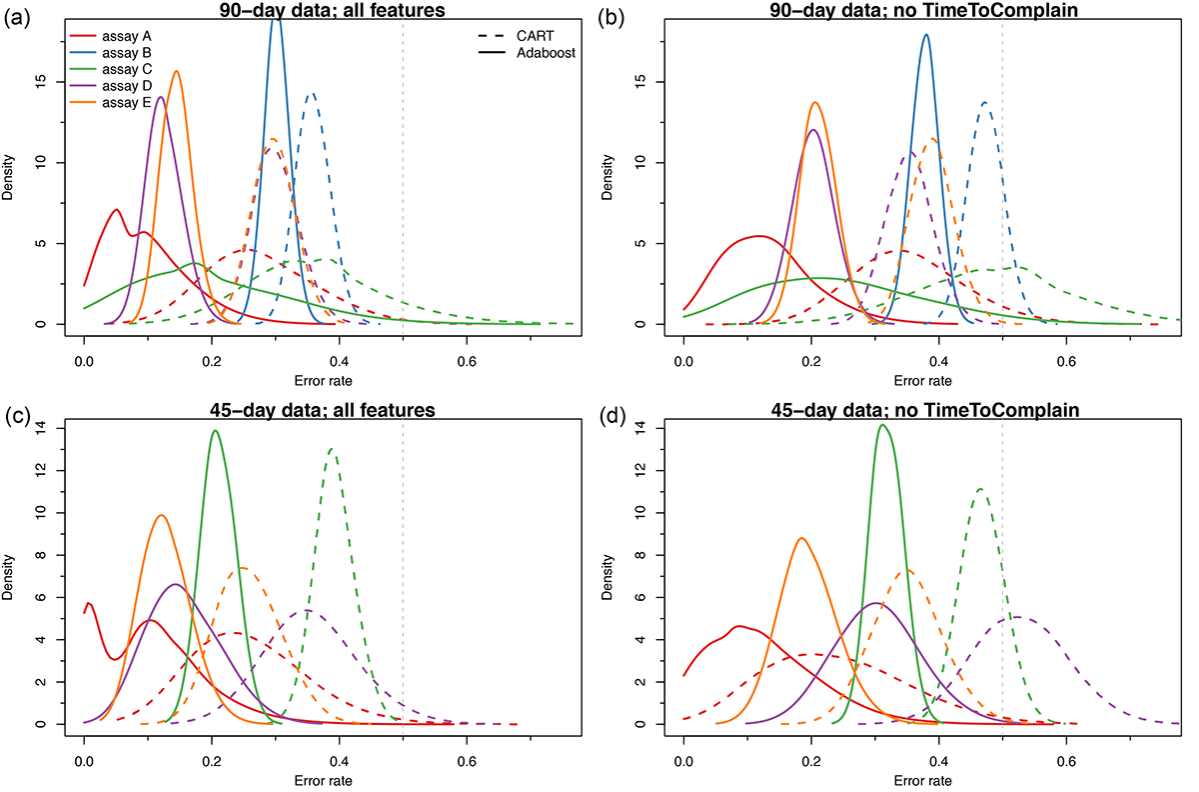
Distribution of prediction error rates for the binned quality check (QC)–only data. Error rates are shown as derived from the cross-validation analyses, where the data were split 2500 times (see Methods). Results are shown for both classifiers, Classification and Regression Trees (CART; broken lines) and adaptive boosting (solid lines), over the five assays considered for the 90-day data with all features (a) or with TimeToComplain removed (b) and likewise for the 45-day data with (c) or not (d) all features. Each assay is color-coded as shown.

In an attempt to denoise the customer data, we first removed non-QC related complaints and trained our classifiers on both the 90- and the 45-day datasets. This led to decreased error rates over all five assays (Multimedia Appendix 8), with some assays benefiting better than others (see assay D vs B) and to similar most important features (Multimedia Appendix 9). Note that for assay C, the small volume of complaints as observed in Figure 3 led to difficulties in training both classifiers on the 45-day data, and results for this assay at this shorter time frame are therefore absent. A closer examination of the confusions tables in this case suggests that no pattern exists in how errors are generated: some assays such as B can fail to predict almost 16% of accuracy high (indicating that the measured concentration is suspected of being higher than the actual value) call areas, whereas others such as E may have a bias in overpredicting QC high (Multimedia Appendix 10). Similarly, binning the QC-high or QC-low data on the QC-only complaints led to further improvements, leading in some cases to classifiers with a zero error rate (eg, see assay A in Figure 3).

**Figure 4:**
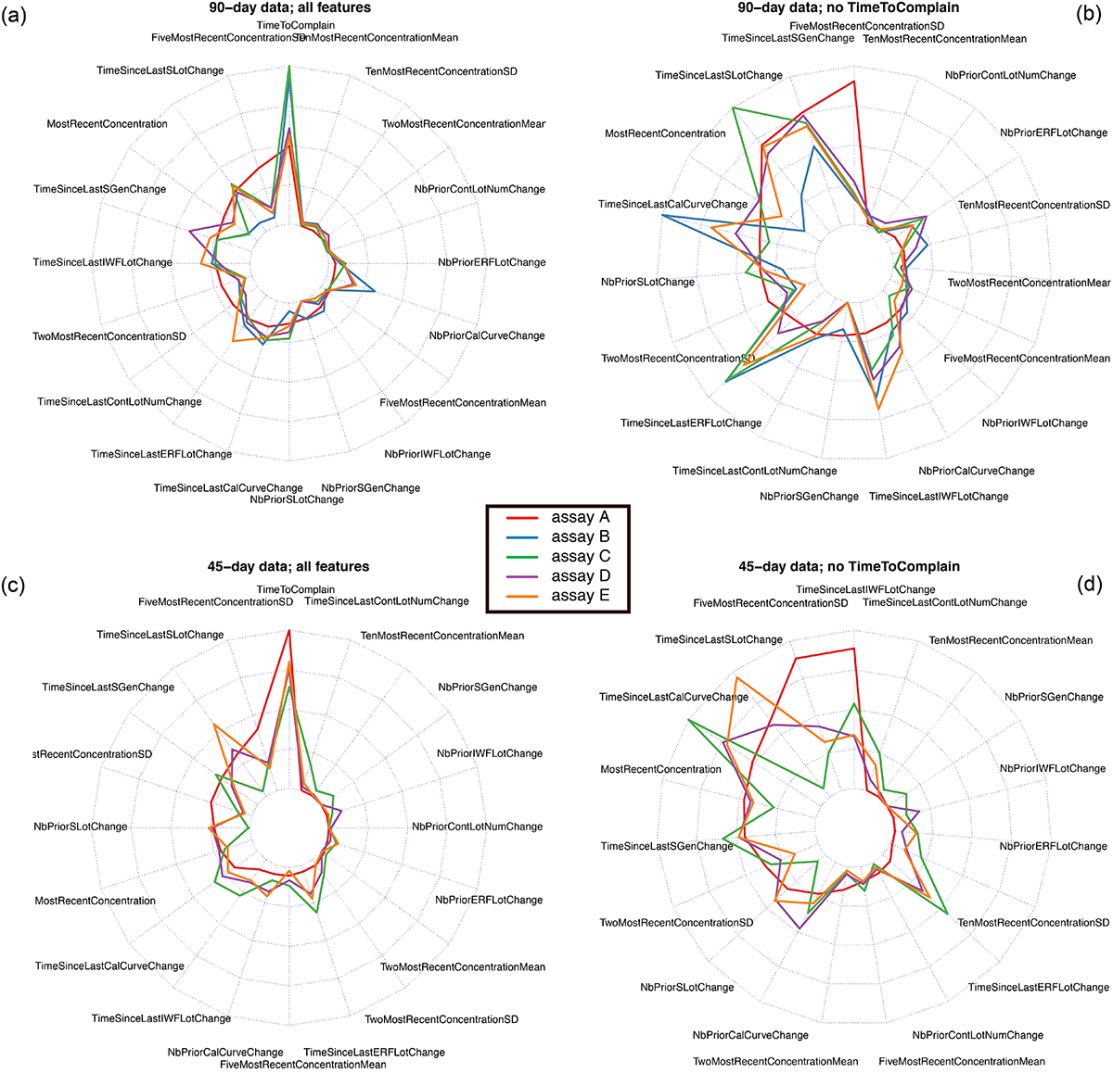
Feature importance under adaptive boosting for the binned quality control (QC)–only data. Importance of the features are shown as radar charts, over the five assays considered. Each assay is color-coded as shown. Top panels are for the whole 90-day datasets, whereas the bottom panels are for the 45-day datasets. Left panels include all feature; right panels exclude TimeToComplain from the models.

In this case, where data are denoised by binning and by only considering QC-only data, the most important features for the classifier based on adaptive boosting remain TimeToComplain for both the 90- and the 45-day datasets (Figure 4). When this feature is removed, timing of events and variability of QC logs remain the most critical factors in determining call areas of customer complaints.

## Discussion

### Principal Findings

Traditionally, failure prediction in industrial applications aims at predicting *when* a particular system is likely to fail [29,30]. Here, we addressed a different question, one not directly related to the timing of failure, but one that focused on which type of failure can be predicted based on customer complaints (Figure 1). This is critical in the health care industry as it can point as to where along the process (from assay production to delivery, to storage, to use, to service on the analyzers) a product issue occurred, and hence, to take remedial steps to avoid further costs—and customer complaints. For this prediction of call areas, we compared two machine-learning algorithms, one based on decision trees (CART) and a more sophisticated one, adaptive boosting, that combines weak classifiers to produce a strong one. We showed that median errors rates can be as low as 8%—while still being as low as 20% in more realistic settings, where it is unknown when a customer is going to complain—and very close to 0% after denoising of the customer data (Figure 2). Note that not knowing when a customer complains does not seem to affect performance order on the five assays tested here.

One of our challenges here is that a complaint is a symptom of an actual product issue. When an issue occurs, the customer may complain, or not. The customer may wait to have several incidences of same issue before complaining, or may choose not to complain because he or she is busy or stopped complaining when it is a recurrent problem. It is also possible that a customer complains when there is no product-related issue. As a result, the complaint database that we used is intrinsically noisy, but (1) This database represents the best data available and (2) The manufacturer’s goal is to improve customer satisfaction by being able to identify issues before (or even without) a complaint call is placed.

To achieve this goal, we resorted to machine learning. As in any machine-learning application—except maybe with some deep-learning applications as those trained directly on images at the pixel level [31]—a key element is the identification and definition of the features used to train a classifier [32,33]. Rather than selecting features in an *ad-ho* *c* manner, as is often the case with EHRs [34], we took inspiration from standard recordings logged by a connected system to identify features that can easily be extracted from the data and that are also likely to reflect QC shifts (Figure 1). This led us to identify two kinds of features: those based on concentration readings and those based on the timing of maintenance events. In the context of this particular manufacturer in the health care industry (Ortho Clinical Diagnostics), we showed that timing of events represent the most important features in predicting a call type in a realistic setting (were the time when a customer complains is unknown), followed by variability in concentration readings (Figure 4). A future improvement of our approach could attempt to perform unsupervised feature learning, as done in deep learning [17]. This might circumvent the following difficulties: different data types (eg, patient health instead of QC data), equipment (eg, Bio-Rad [Hercules, California] rather than Ortho Clinical Diagnostics), or logging system (eg, Bio-Rad’s UnityConnect vs e-Connectivity), which might require the definition of alternative features. However, it is likely that (1) All connectivity solutions log similar chemistry end points (concentrations, timing of service, etc) and that (2) Sophisticated machine-learning algorithms such as adaptive boosting will still produce quite impressive results. Here, we did not evaluate other algorithms such as support vector machines [14], neural networks [13] or deep learning [17], or others, as most of these approaches have the same goals and can behave equally well [35].

### Limitations

Some additional questions and limitations remain, however. First, we extracted data for a period of 90 days and showed that the length of this period could affect performances. Indeed, shorter training periods seem to improve prediction performances when complaint rate is high. If complaint volume does affect performance, the length of the period used for analytics should be optimized in real time. This point was not addressed here and will require further investigation, in particular, to better understand the link between the volume of customer complaints for specific call areas, the features that become the most important, and how prediction performances are affected (Figure 4). Second, we only focused on five assays and showed that our general approach seems to deliver similar performances across those particular assays. However, this need not be the case, and a systematic survey should be undertaken. Third, we employed only one particular system here, the VITROS System, manufactured by Ortho Clinical Diagnostics. However, it is not immediately clear whether our approach can be ported to other systems, be they distributed by the same or by other manufacturers. Yet, it may be expected that most systems from most manufacturers will log QC data in a similar way, which can be interpreted in the same way as here (see feature definitions). Fourth, we were limited in our analysis of QC data by not having access to actual verifiers from the manufacturer. This forced us to resort to changes in the measured concentrations, rather than simply checking departures between measurements and verifiers. However, obtaining these data was in our case challenging, as these data were only available as individual PDF files for each performance verifier lot (there were hundreds of lots). Obtaining information about these verifiers would help train our predictive tools. Fifth, we exclusively focused on QC data used for monitoring health care systems, not on patients’ health. This was done to avoid complications linked to obtaining consent forms from patients in hospitals that are themselves scattered around the world. Eventually, health care analytics should also monitor individual patients and hence, help physicians in their diagnosis. Sixth, call areas, which we aimed at predicting, are used by a manufacturer to identify an issue with the product or with the analyzer in the complaint handling process: they are not the root cause of the issue, which can only be determined through what is known as a root cause investigation (RCI). RCIs are, however, very time consuming to conduct, especially on analyzers that are distributed globally, so that most of the time, the actual cause of a reported issue may not be known. However, knowing which issue may arise (ie, our prediction of call areas) instead of the actual cause can help manufacturers to initiate targeted RCIs more proactively. Finally, we have only presented one side of the health care analytics in predicting call areas, not *when* failures occur. An integrated solution should put together both questions, possibly by merging our approach with traditional time series methods [29,30].

In the future, a more agnostic approach with respect to feature definition may be required: indeed, the features that are based on concentration readings all depend, to some extent, on the exact time when a customer complained. This time is unknown when performing real-time analytics. To circumvent this limitation, it might be better to implement a sliding window, defined over a time period *T*=[*t*_0_*, t*_1_], and use time *t*=*t*_1_ as the cutoff point to define features that are based on the timing of events.

### Conclusions

Although the approach we described will require further validation and testing, the ultimate goal is to implement this kind of predictive tool into the global monitoring system of IVD analyzers to help manufacturers be more proactive in detecting quality issues of the various assays they marketed around the world. This may help them pinpoint where in the manufacturing process issues are likely to originate—eg, if only a particular lot number is globally generating the same call area, a manufacturing problem specific to this lot can be identified. As such, we might one day be able to develop *automate* *d analytics* or systems that can not only identify when and how failures will occur but also automatically take remediation steps to resolve these issues, in real time, without the intervention of any human being [5].

In the meantime, the US FDA is planning to use big data to guide regulatory decisions [24]. Consequently, medical companies will soon have to harness all the data logged by their instruments and use these data to their full potential to further improve the health care system. Our contribution here is a first step in this direction, laying ground to predicting call areas, and hence, enabling manufacturers, the expected end user of our approach, to be more proactive in postmarket surveillance. We predict that by combining our machine-learning approach with traditional time series analysis, we will eventually be able to predict when a customer will complain, in addition to what he or she will complain about. This work paves the way to developing an automated tool to anticipating customer complaints and identifying product quality issues through connected systems.

## Appendix

Multimedia Appendix 1 Detailed description of the e-Connectivity features.

Multimedia Appendix 2 Detailed description of the customer features.

Multimedia Appendix 3 Description of the 99 error codes reported by the analyzers over the five assays.

Multimedia Appendix 4 Distribution of mean concentration reading per sample for the same assay. For each sample in the e-Connectivity data, the mean of all concentration readings was taken, and their distribution over the entire e-Connectivity 90-day data set was plotted. This distribution is multimodal; modes were estimated and are shown as vertical red dotted lines.

Multimedia Appendix 5 Distribution of prediction error rates for the unfiltered customer data. Error rates are shown as derived from the cross-validation analyses, where the data were split 2500 times (see Methods). Results are shown for both classifiers, CART (broken lines) and adaptive boosting (solid lines), over the five assays considered, for the 90-day data with all features (a) or with TimeToComplain removed (b), and likewise for the 45-day data with (c) or not (d) all features. Each assay is color-coded as shown.

Multimedia Appendix 6 Distribution of call areas for each assay. Distributions are shown for the whole 90-day data sets (a) and the 45-day data set (b). Each assay is color-coded as shown. Non-QC related call areas were filtered out.

Multimedia Appendix 7 Feature importance under adaptive boosting for the unfiltered customer data. Importance of the features are shown as radar charts, over the five assays considered. Each assay is color-coded as shown. Top panels are for the whole 90-day data sets, while the bottom panels are for the 45-day data sets. Left panels include all feature, right panels exclude TimeToComplain from the models.

Multimedia Appendix 8 Distribution of prediction error rates for the QC-only customer data. Error rates are shown as derived from the cross-validation analyses, where the data were split 2500 times (see Methods). Results are shown for both classifiers, CART (broken lines) and adaptive boosting (solid lines), over the five assays considered, for the 90-day data with all features (a) or with TimeToComplain removed (b), and likewise for the 45-day data with (c) or not (d) all features. Each assay is color-coded as shown.

Multimedia Appendix 9 Feature importance under adaptive boosting for the QC-only customer data. Importance of the features are shown as radar charts, over the five assays considered. Each assay is color-coded as shown. Top panels are for the whole 90-day data sets, while the bottom panels are for the 45-day data sets. Left panels include all feature, right panels exclude TimeToComplain from the models.

Multimedia Appendix 10 Examples of confusion tables obtained during cross-validation on the 90-day data, filtered for quality control (QC)–only call areas (data not binned by QC level). Numbers on the diagonal show accurate predictions; false predictions are below the diagonal, whereas missed predictions are above.

## Abbreviations

CART: Classification and Regression Trees
EHR: electronic health record
FDA: Food and Drug Administration
IVD: in vitro diagnostic
QC: quality control
RCI: root cause investigation
