# Supplementary material for: Predicting the Reasons of Customer Complaints: A First Step Toward Anticipating Quality Issues of In Vitro Diagnostics Assays with Machine Learning
Source: JMIR Med Inform. 2018 May 15;6(2):e34. doi: 10.2196/medinform.9960 (PMC5974458; doi:10.2196/medinform.9960)
Supplement: Multimedia Appendix 1 [file medinform_v6i2e34_app1.pdf]

Data	Abbreviation	Short description
E-conn	Assay	abbreviation of assay name (recoded here).
	J Number	unique identifier assigned to each analyzer placed.
	F Concentration	concentration of solute (Assay); QC result.
	Units	unit of measured concentration (mmol/L)
	F Concentration (SI)	concentration of solute (Assay); QC result.
	Units SI	unit of measured concentration (SI).
	Reagent Lot Number	reagent lot number.
	S Gen	manufacturing generation number: generation (Gen) is a term used to describe those slide lots that can use the same Supplementary Assigned Values (SAVs) for calibration. Slide lots from the same generation have the same Range of Means on the assay sheet. The Gen number for each lot of slides is found on the packaging as well as on the side and top labels of individual slide cartridges.
	S Lot	manufacturing lot number: a lot is a specific identified unit produced in a unit of time or quantity in a manner that assures it is having uniform character and quality within specified limits.
	ERF Lot	Electrolyte Reference Fluid (ERF) is used in the potentiometric measurement of ion-selective electrode slides (ISE): sodium (Na^{+}), potassium (K^{+}), and chloride (Cl^{-}). Calibration is needed when changing new lot of ERF.
	IWF Lot	Immuno-Wash Fluid (IWF) lot number: calibration is needed when changing new lot of immuno-wash.
	Control Lot Number	performance verifier lot number: Calibration Verification is a means of assessing whether a calibration is acceptable by using results from each level of Performance Verifiers. At a minimum, these results should be within the ROM for that control.
	Cal Curve ID	calibration curve ID. Calibration Curve is a method in analytical chemistry, used to determine or measure the concentration of a particular substance in a sample. This is done by comparing this sample of unknown concentration to a set of standard samples whose concentration is known.
	Result ID	unique identifier (encrypted) of QC result.
	Sample Name	unique identifier (encrypted) of sample, which corresponds to a particular QC run.
	Time Metering	timestamp of concentration log through E-conn.
	Total Dilution	dilution factor.
	Operator Dilution	operator requested dilution.
	Body Fluid	fluid type (serum, plasma or urine).
