# Supplementary material for: Predicting the Reasons of Customer Complaints: A First Step Toward Anticipating Quality Issues of In Vitro Diagnostics Assays with Machine Learning
Source: JMIR Med Inform. 2018 May 15;6(2):e34. doi: 10.2196/medinform.9960 (PMC5974458; doi:10.2196/medinform.9960)
Supplement: Multimedia Appendix 2 [file medinform_v6i2e34_app2.pdf]

customer	Create Audit Date	timestamp of when complaint was placed.
	Call Subject	same as Assay in E-conn: it is the product name.
	Call Area	classification of concern or problem of the product or the analyzer-generated condition.
	Resolution	code describing how the complaint was resolved.
	Complaint Number	unique identifier of each complaint.
	Customer Number	unique identifier of each customer.
	J Number	analyzer serial number.
	Lot number	reagent lot number.
	Region	geographic region where complaint was placed.
	Call Status	current call status of complaint (Closed or Open).
	Problem description	free-text field describing the complaint.
