# Supplementary material for: Predicting the Reasons of Customer Complaints: A First Step Toward Anticipating Quality Issues of In Vitro Diagnostics Assays with Machine Learning
Source: JMIR Med Inform. 2018 May 15;6(2):e34. doi: 10.2196/medinform.9960 (PMC5974458; doi:10.2196/medinform.9960)
Supplement: Multimedia Appendix 3 [file medinform_v6i2e34_app3.pdf]

Call Area	Call Area Description
ACCH	ACCURACY HIGH: measured concentration is suspected of being higher than the actual value
ACCL	ACCURACY LOW: measured concentration is suspected of being lower than the actual value
CAL	CALIBRATION
CORR	CORRELATION
DAMAGED	DAMAGED PARTS/COMPONENTS
DISCRES	DISCREPANT RESULTS
EMTYCNTR	EMPTY CONTAINER
LEAKSPIL	LEAKAGE/SPILLAGE
LINEAR	LINEARITY
OTHER	OTHER
OUTLIERH	OUTLIER HIGH: outlier results that are higher than expected
OUTLIERL	OUTLIER LOW: outlier results that are lower than expected
PREC	PRECISION
PROF	PROFICIENCY
QCDH	QC DRIFT HIGH: Quality control measured concentration is drifting high
QCDL	QC DRIFT LOW: Quality control measured concentration is drifting low
QCH	QC HIGH: Quality control measured concentration is suspected of being higher than the actual value
QCL	QC LOW: Quality control measured concentration is suspected of being lower than the actual value
QCSH	QC SHIFT HIGH: Quality control measured concentration is shifting high
QCSL	QC SHIFT LOW: Quality control measured concentration is shifting low
REPCONF	REPORT CONFIGURATION
Z502-004	MACHINE CODE
Z506-001	MACHINE CODE
Z506-020	MACHINE CODE
Z506-031	MACHINE CODE
Z523-017	MACHINE CODE
Z541-032	MACHINE CODE
Z542-030	MACHINE CODE
Z542-032	MACHINE CODE

Call Area	Call Area Description
Z542-036	MACHINE CODE
Z542-037	MACHINE CODE
Z542-038	MACHINE CODE
Z546	MACHINE CODE (TEST PROCESSING * INITIALIZATION * slide unavailable)
Z547	MACHINE CODE (TEST PROCESSING * RESPONSE MANAGEMENT * invalid data)
Z54L	MACHINE CODE (TEST PROCESSING * RESPONSE MANAGEMENT * calibrator error)
Z551-008	MACHINE CODE
Z6B6	MACHINE CODE
Z6B9	MACHINE CODE (PF-PREDICTION * ELECTROMETER * invalid reading)
Z6BG	PF-PREDICTION * ELECTROMETER results outside limit
Z6F2	MACHINE CODE (CALIBRATION * replicated slide responses exceed range)
Z6FH	MACHINE CODE (CALIBRATION * invalid reading)
Z6H3	MACHINE CODE
Z6LU	IR WASH DETECTION * IR wash failed
Z6M9	MACHINE CODE (CALIBRATION * parameters failed extrema test)
Z7EA	MACHINE CODE
ZA03-095	MACHINE CODE
ZEAU	SAMPLE METERING * WETNESS DETECTOR * No slide present
ZJ01-032	MACHINE CODE
ZJ01-039	MACHINE CODE
ZJ01-064	MACHINE CODE
ZJ22	MACHINE CODE
ZMB6-311	WELL DISPENSE SCREW DISPENSE W
ZMB6-31A	WELL DISPENSE SCREW DISPENSE W
ZMB9-310	WELL DISPENSE SCREW PREPOSITIO
ZMB9-31C	WELL DISPENSE SCREW PREPOSITIO
ZMBQ-100	MACHINE CODE
ZMF6-4LB	MWRM DISPENSE OUTER BUBBLE
ZMFM-41A	MACHINE CODE

Call Area	Call Area Description
ZMFM-42A	MACHINE CODE
ZPES-603	FINAL WW - WASH %D VOLUME EXCE
ZPES-608	FINAL WW - TWO CONSECUTIVE SAM
ZPES-609	FINAL WW - VOLUME VERIFICATION
ZPU1-209	SLIDES COULD NOT BE RESERVED F
ZPU1-229	UWELL REAGENT COULD NOT BE RES
ZPU1-233	NO RESERVATION FOR %S: NO CALI
ZPV7-033	SHELF EXP DATE MISSING FOR LOT
ZPV8-047	LEVEL IN PACK INNER BOTTLE HAS
ZPV8-054	LEVEL IN INNER BOTTLE HAS DECR
ZPVD-011	MACHINE CODE
ZPVD-012	REAGENT %S HAS EXPIRE
ZPVD-014	EXPIRATION UNKNOWN FOR REAGENT
ZPW1-418	SAMPLE VISCOSITY HIGH
ZPW6-024	AN IN-PROCESS CALIBRATION FAIL
ZPW7-012	CALIBRATOR SIGNAL INDEX ABOVE
ZPW7-015	CALIBRATOR DELTA BETWEEN LEVEL
ZPW7-017	CALIBRATOR DELTA BETWEEN LEVEL
ZPW7-018	CALIBRATOR DELTA BETWEEN LEVEL
ZPW7-019	CALIBRATOR DELTA BETWEEN LEVEL
ZPW8-017	MACHINE CODE
ZPW8-018	MACHINE CODE
ZPW8-037	SLIDE VALIDATION READ WAS OUT
ZPY2-020	CAL RULES REMOVED ALL ASSAYS F
ZPZ0-095	ADD DATA MISSING OR INCORRECT
ZS88-107	MACHINE CODE
ZTA4-40E	SS1 - UNREADABLE BARCODE SLOT
ZTJ5-43A	MACHINE CODE
ZTJ6-41A	WF METERING PRESSURE ERROR

Call Area	Call Area Description
ZTM5-45B	2°/UIA METER ASP BUBBLE
ZTM5-45G	2°/UIA METER ASP INSUFF FLUID
ZTM5-45H	UIA METERING ASPIRATE ID:%S PO
ZTMH-4MA	UIA METERING MIX MIDDLE RING
ZU90-320	RESULT OUTSIDE IMPEDANCE LIMIT
ZU90-321	REPLICATES OUT OF RANGE ID: %S
ZU90-322	REPLICATES OUT OF RANGE ID: %S
ZU90-323	REPLICATES OUT OF RANGE ID: %S
ZU90-326	CALIBRATION - INVALID READING
ZU90-350	RESPONSE HIGH
ZU90-351	RESPONSE BELOW SPLINE RANGE ID
ZU90-382	IR WASH ERROR ID: %S ASSAY:
