# Supplementary figures and images for: Predicting the Reasons of Customer Complaints: A First Step Toward Anticipating Quality Issues of In Vitro Diagnostics Assays with Machine Learning
Source: JMIR Med Inform. 2018 May 15;6(2):e34. doi: 10.2196/medinform.9960 (PMC5974458; doi:10.2196/medinform.9960)

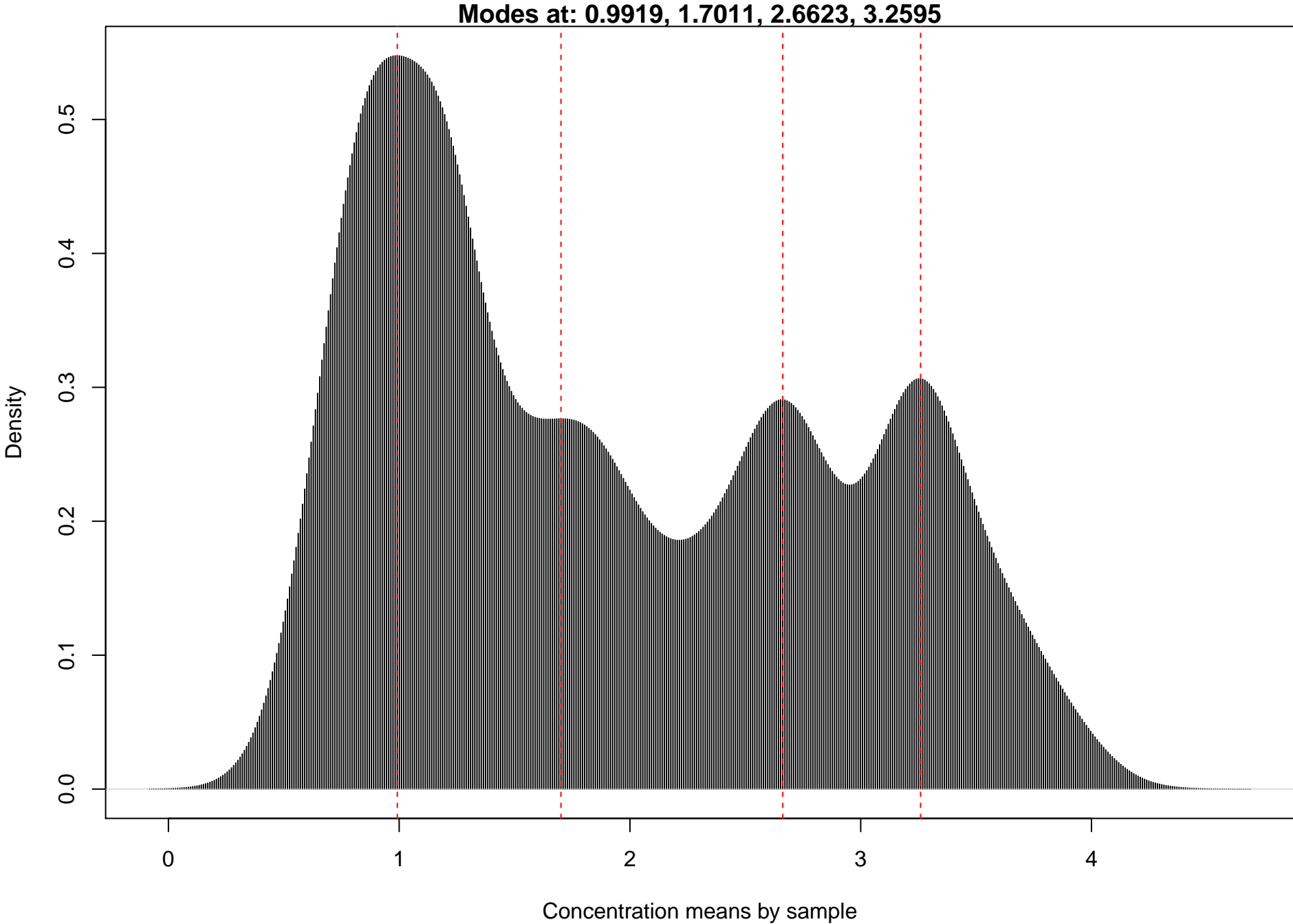

Supplement: Multimedia Appendix 4 [file medinform_v6i2e34_app4.pdf]

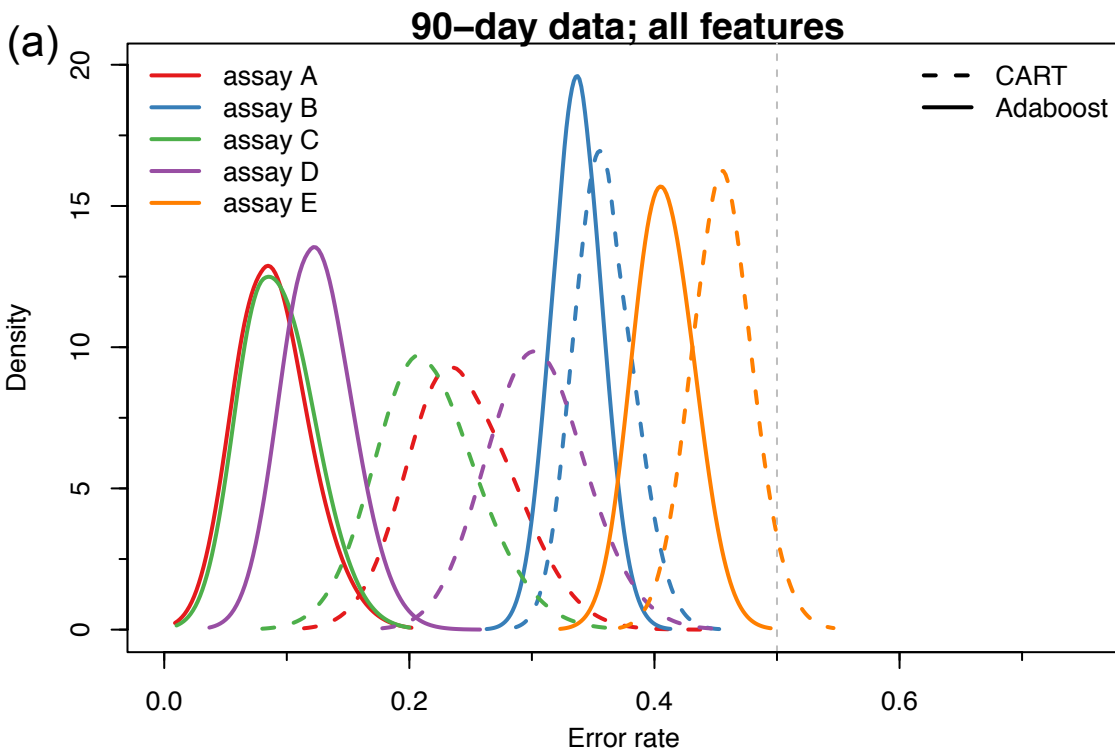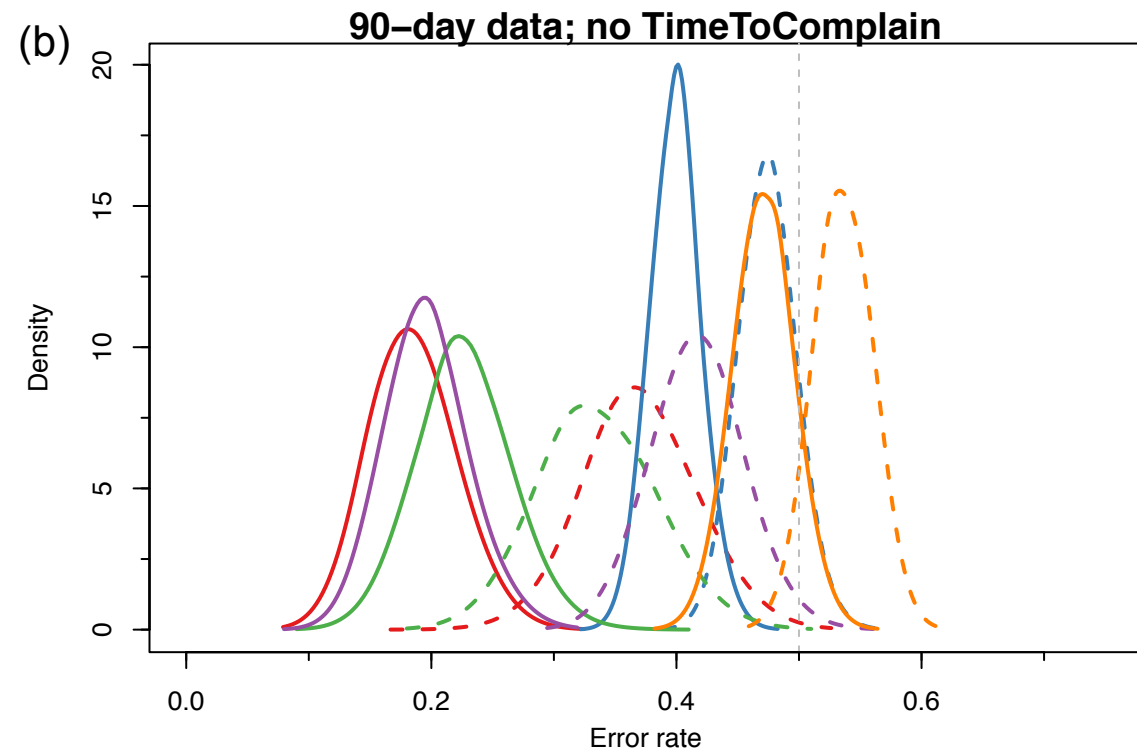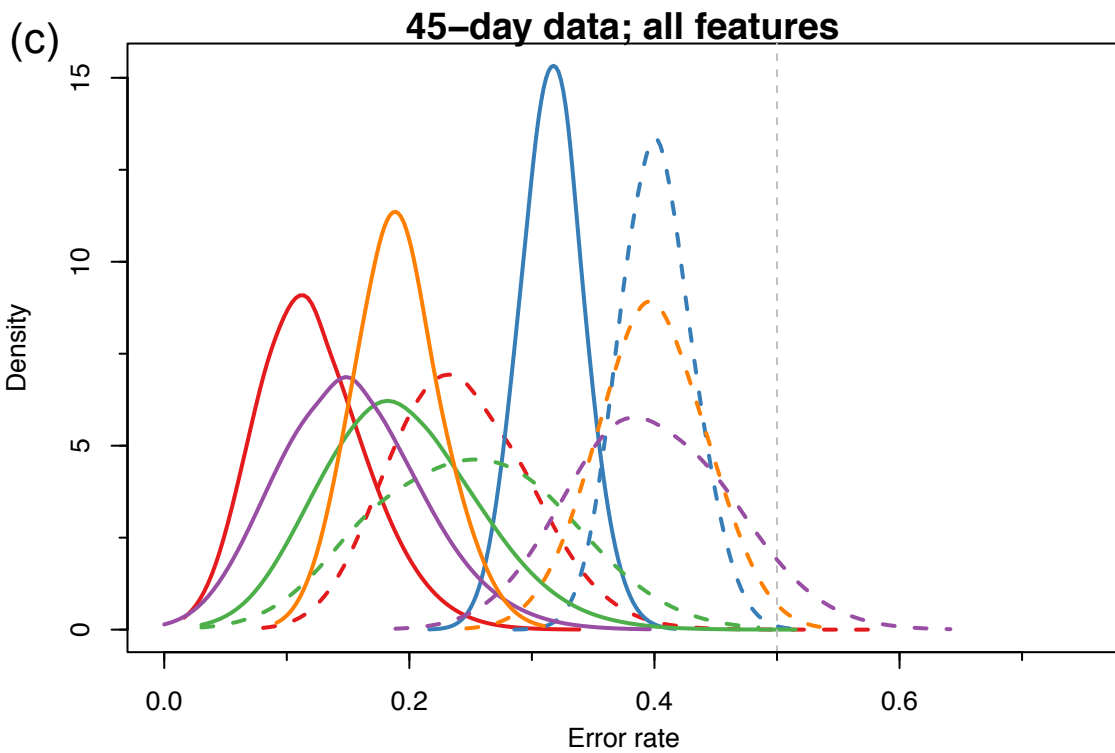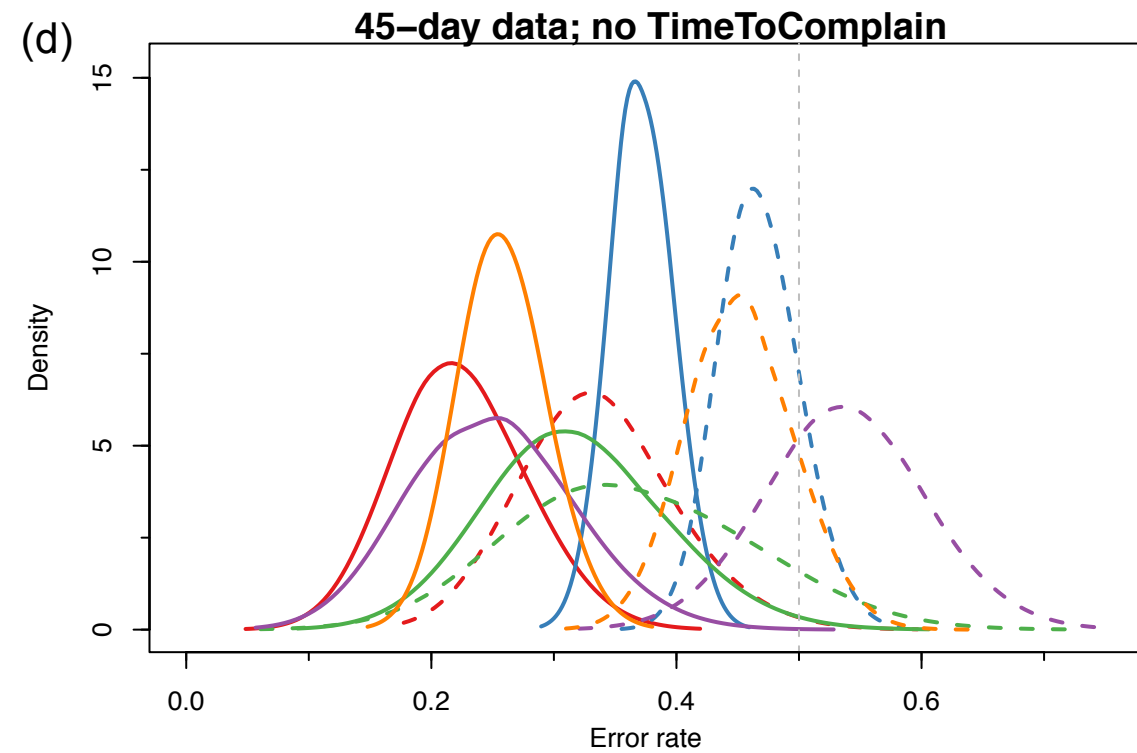

Supplement: Multimedia Appendix 5 [file medinform_v6i2e34_app5.pdf]

(a)

90-day data

Number of complaints

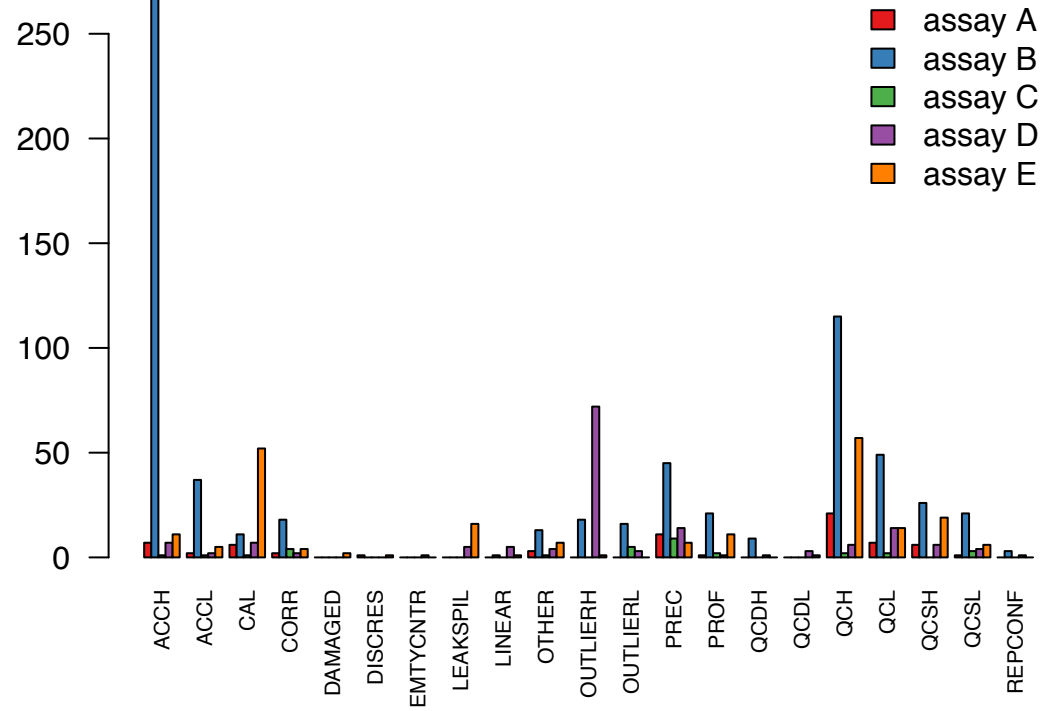

(b)

45-day data

Number of complaints

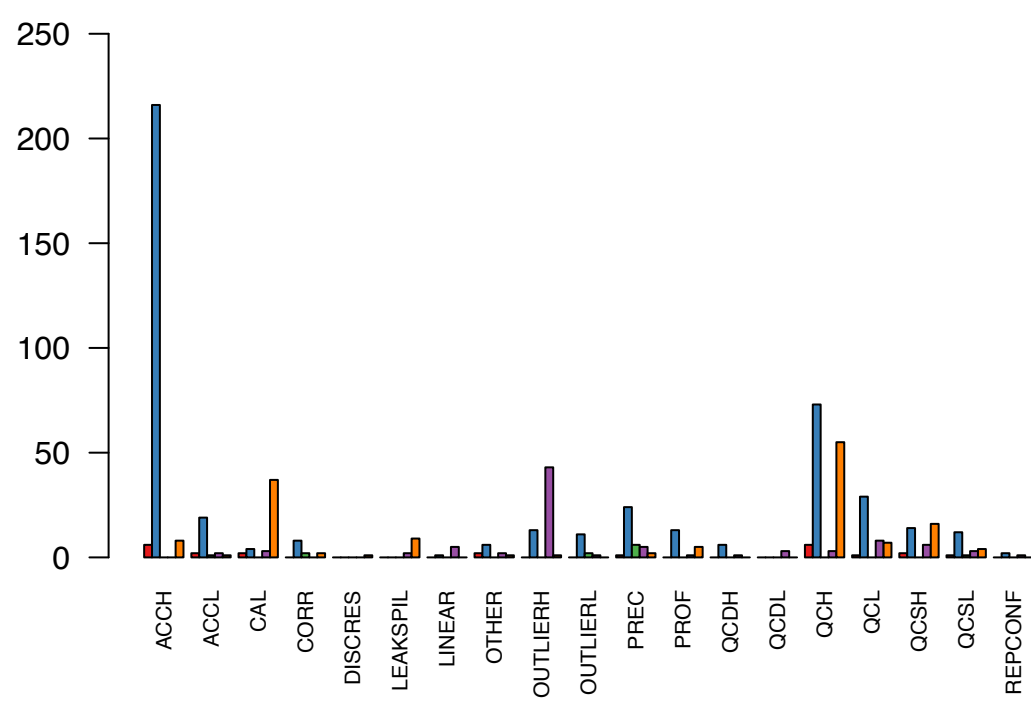

Supplement: Multimedia Appendix 6 [file medinform_v6i2e34_app6.pdf]

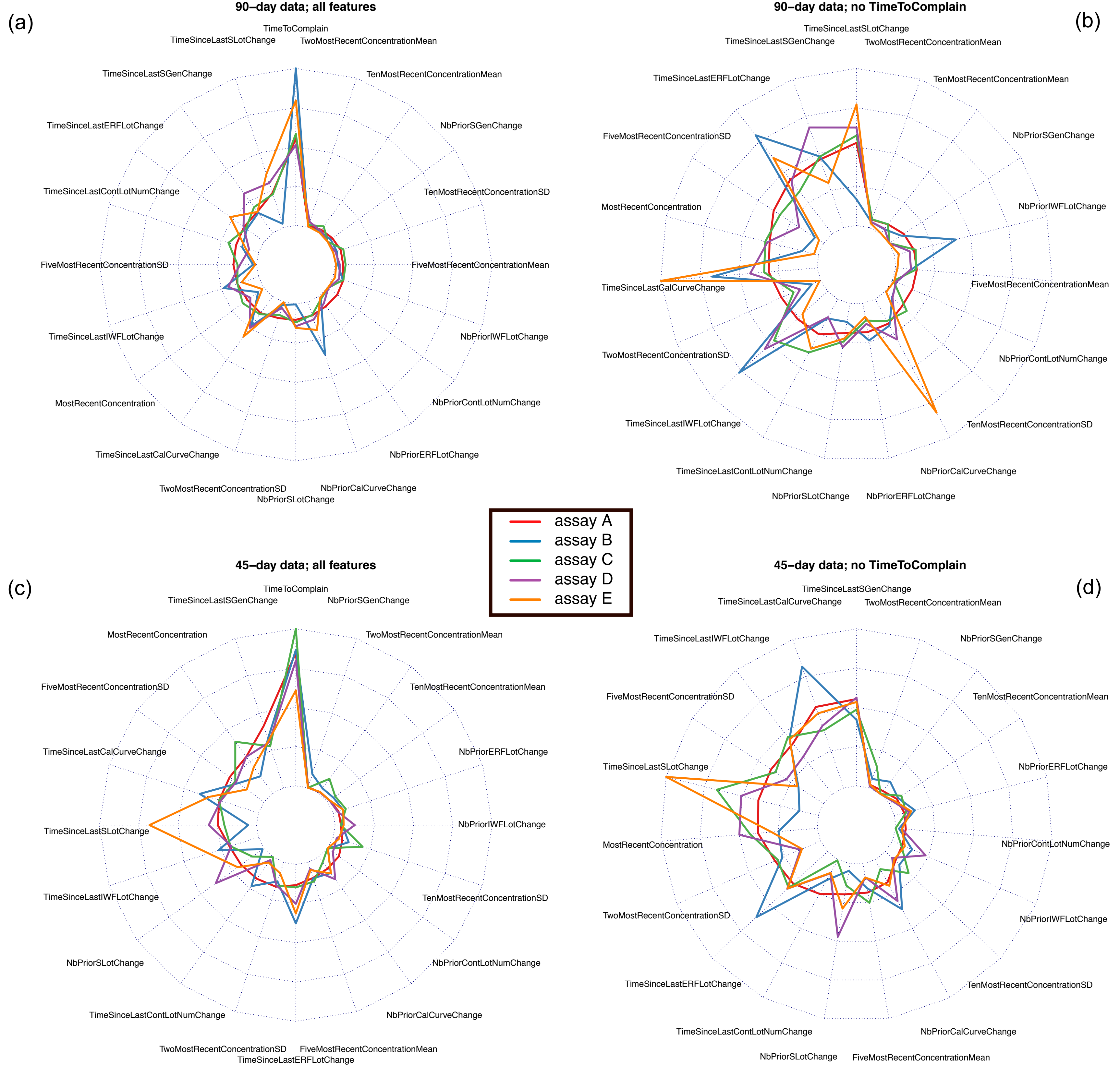

Supplement: Multimedia Appendix 7 [file medinform_v6i2e34_app7.pdf]

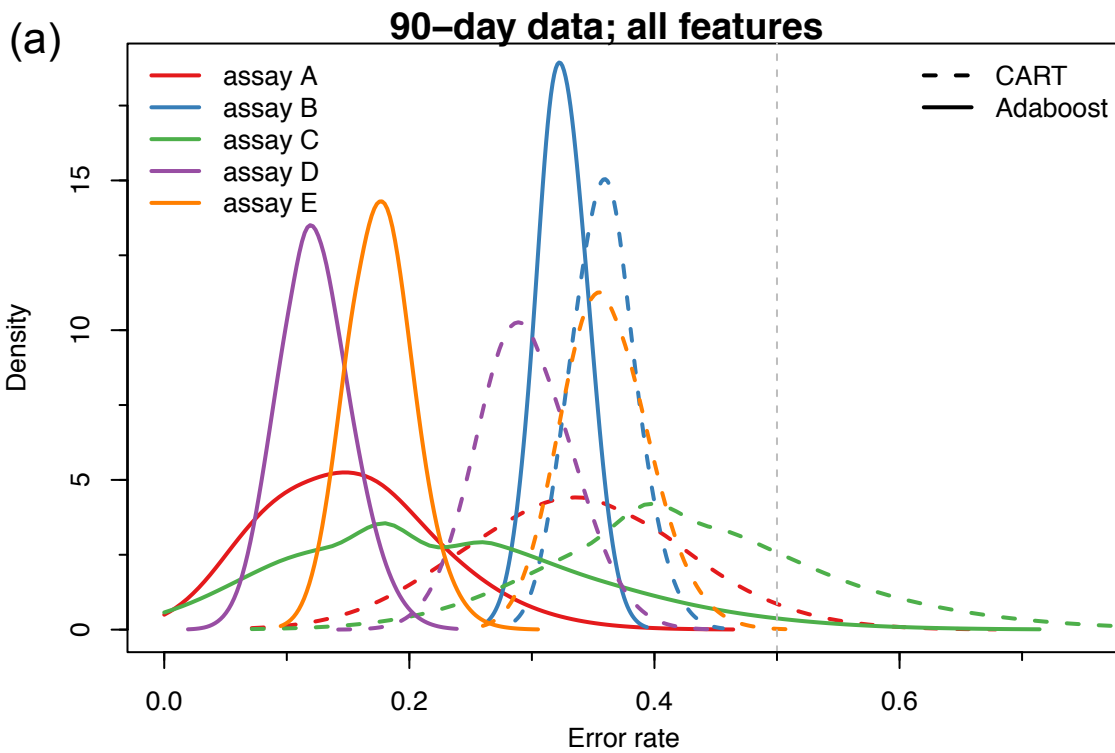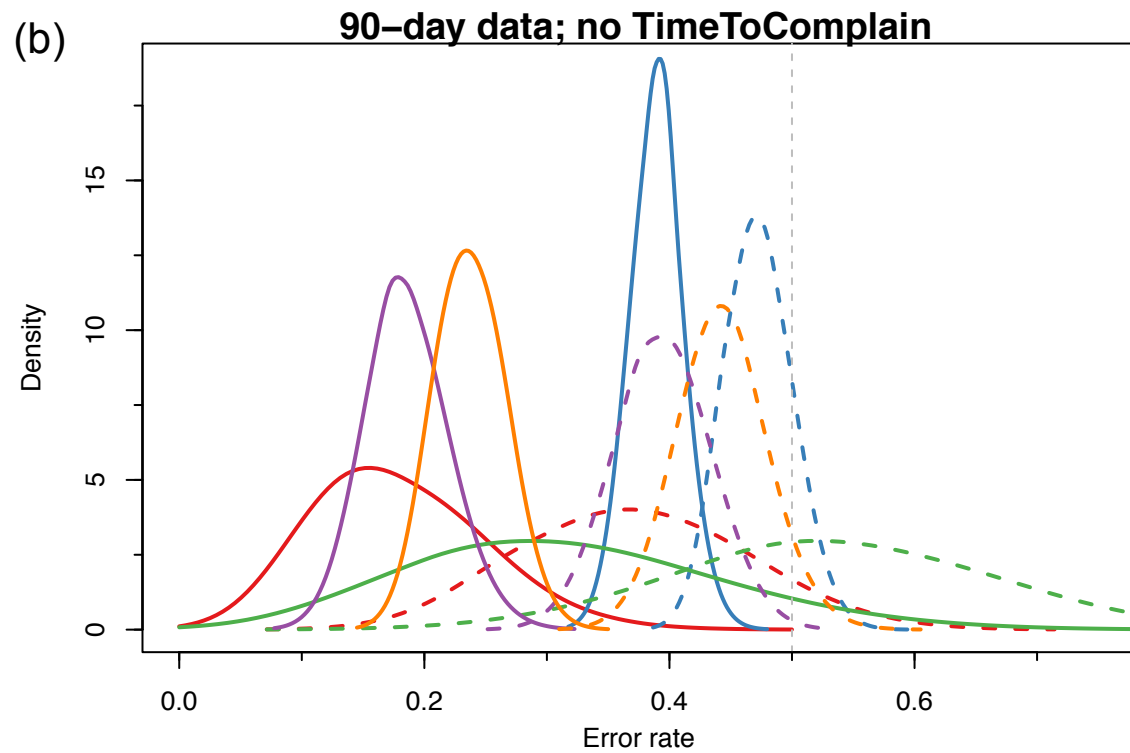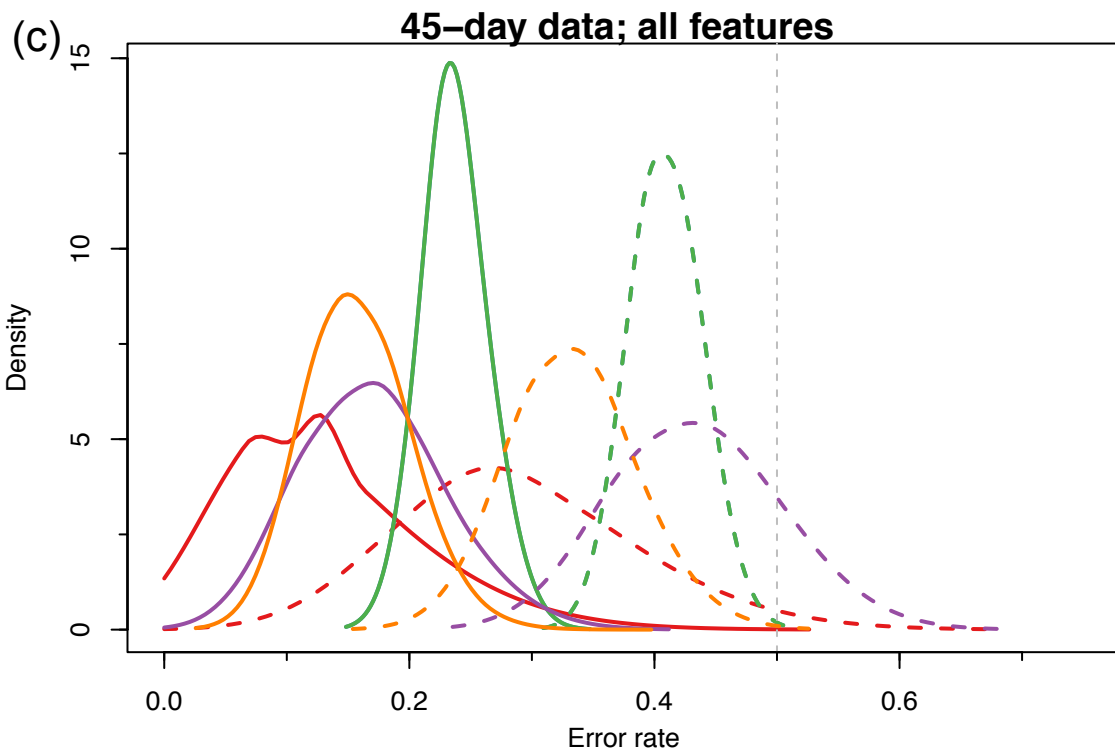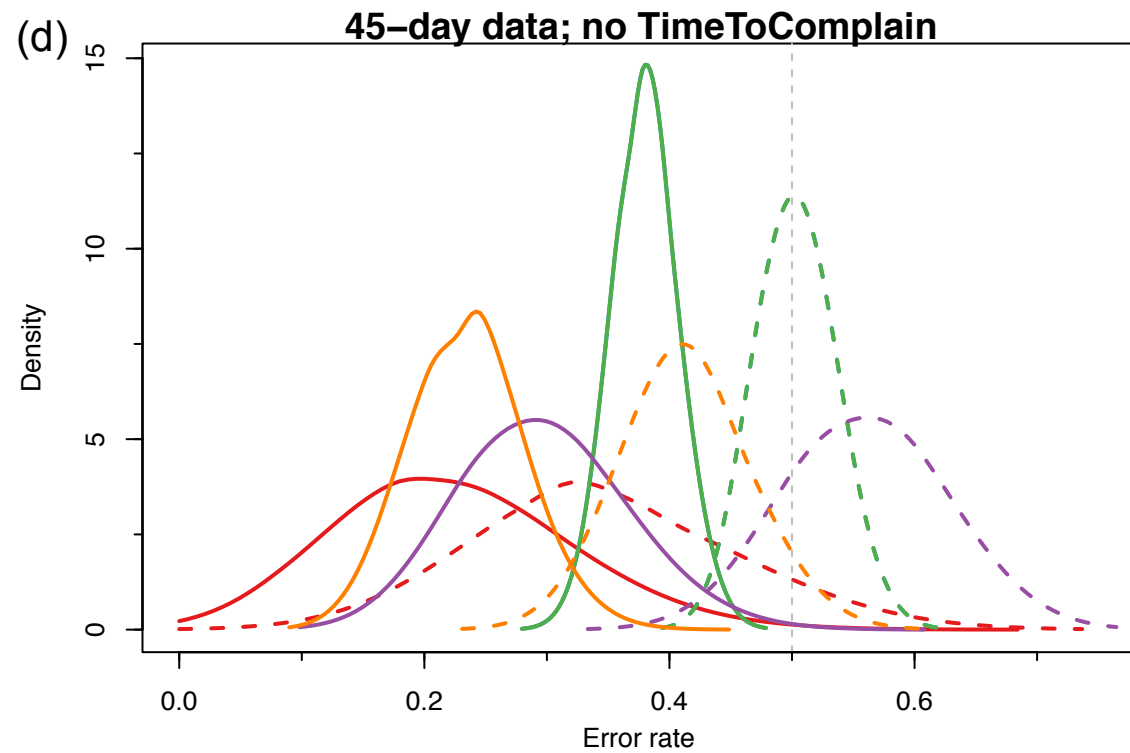

Supplement: Multimedia Appendix 8 [file medinform_v6i2e34_app8.pdf]

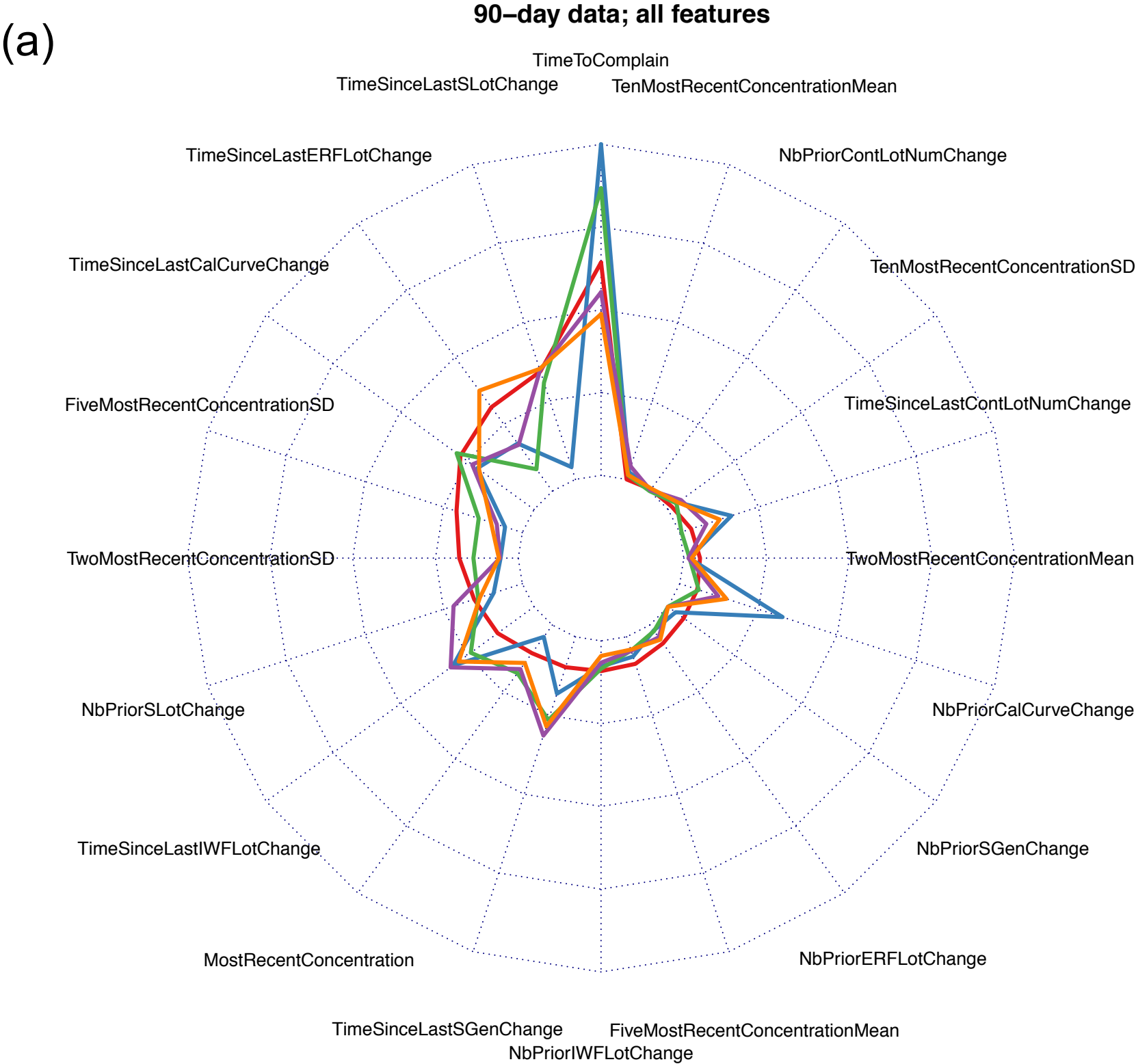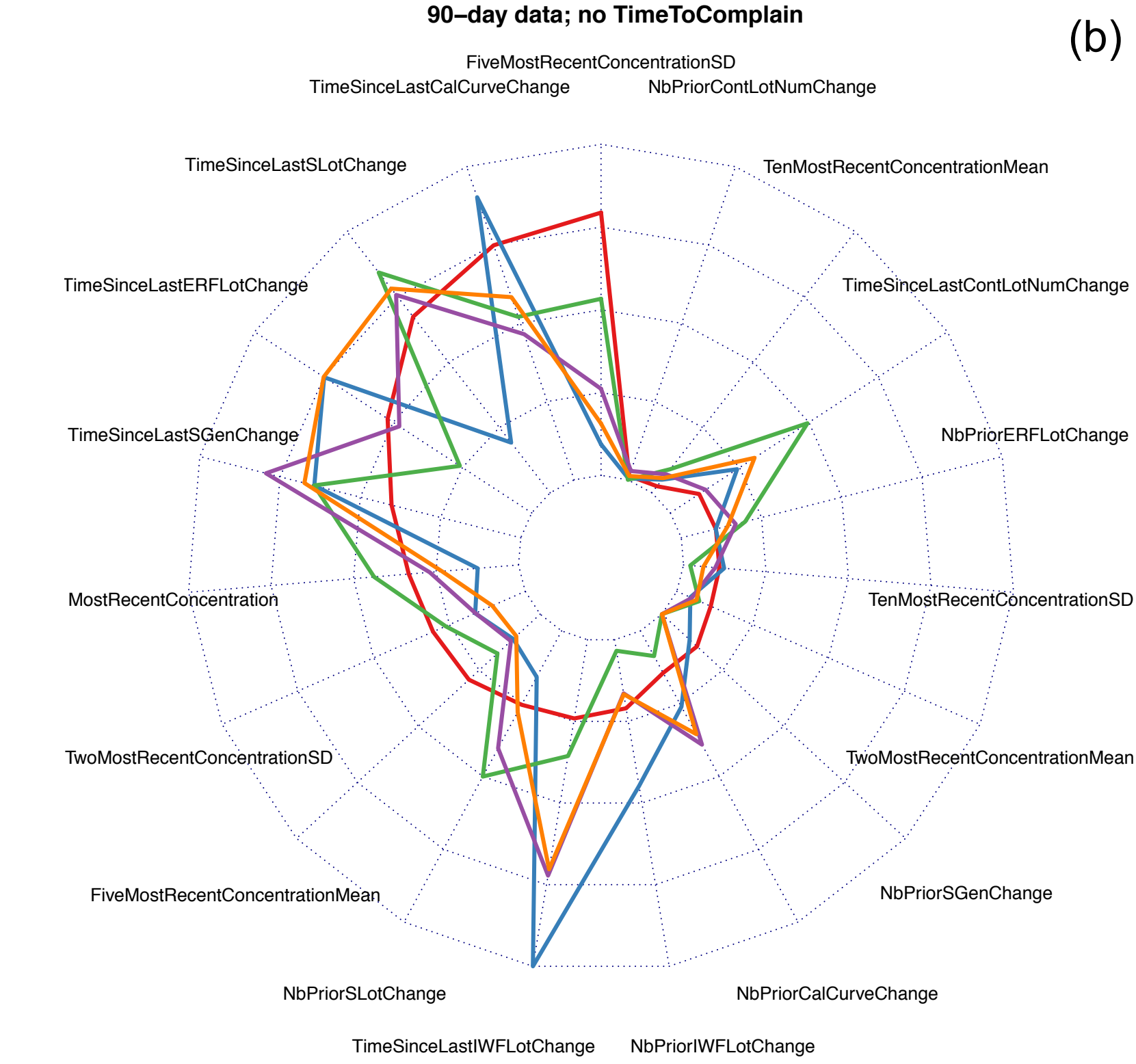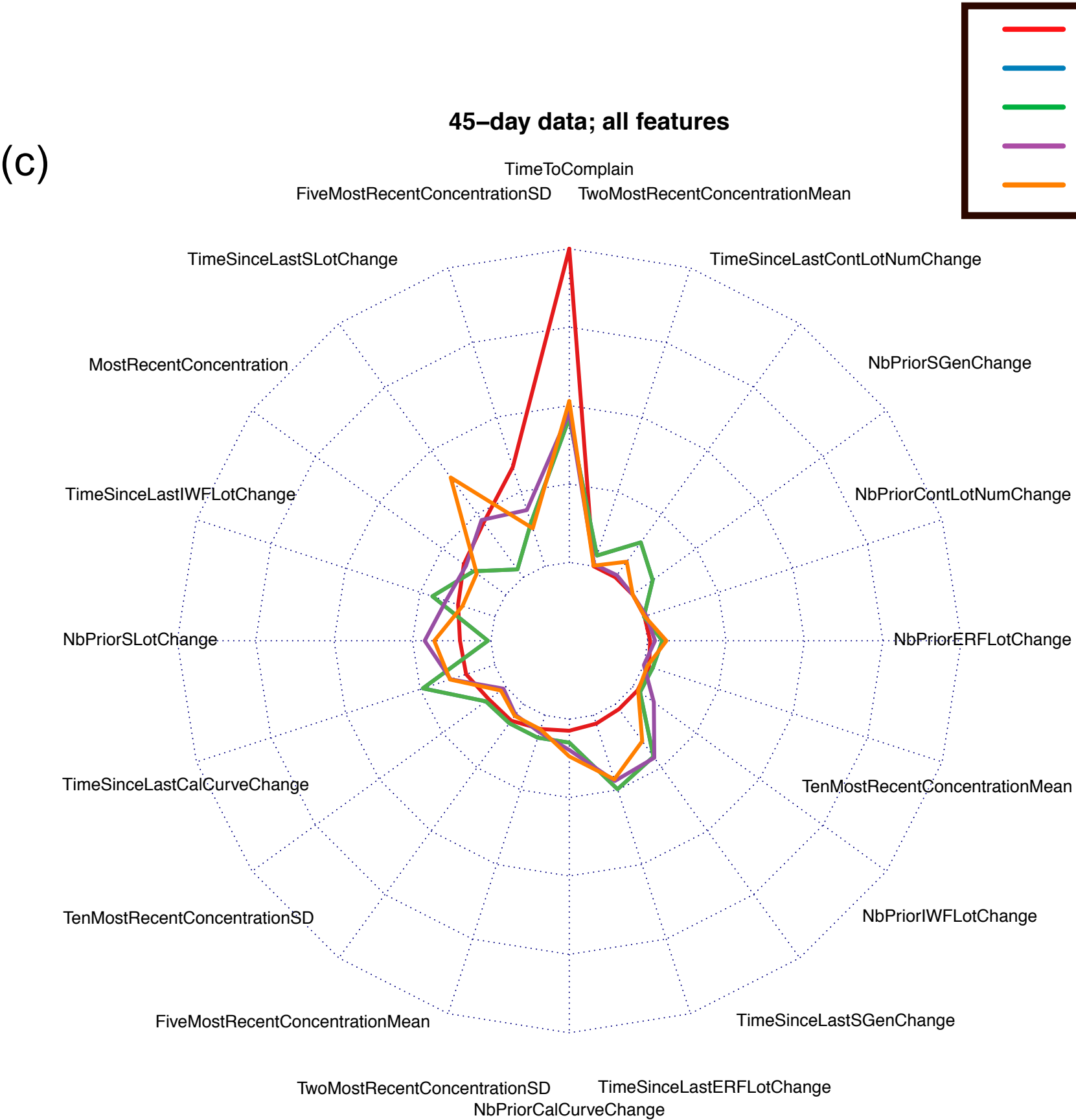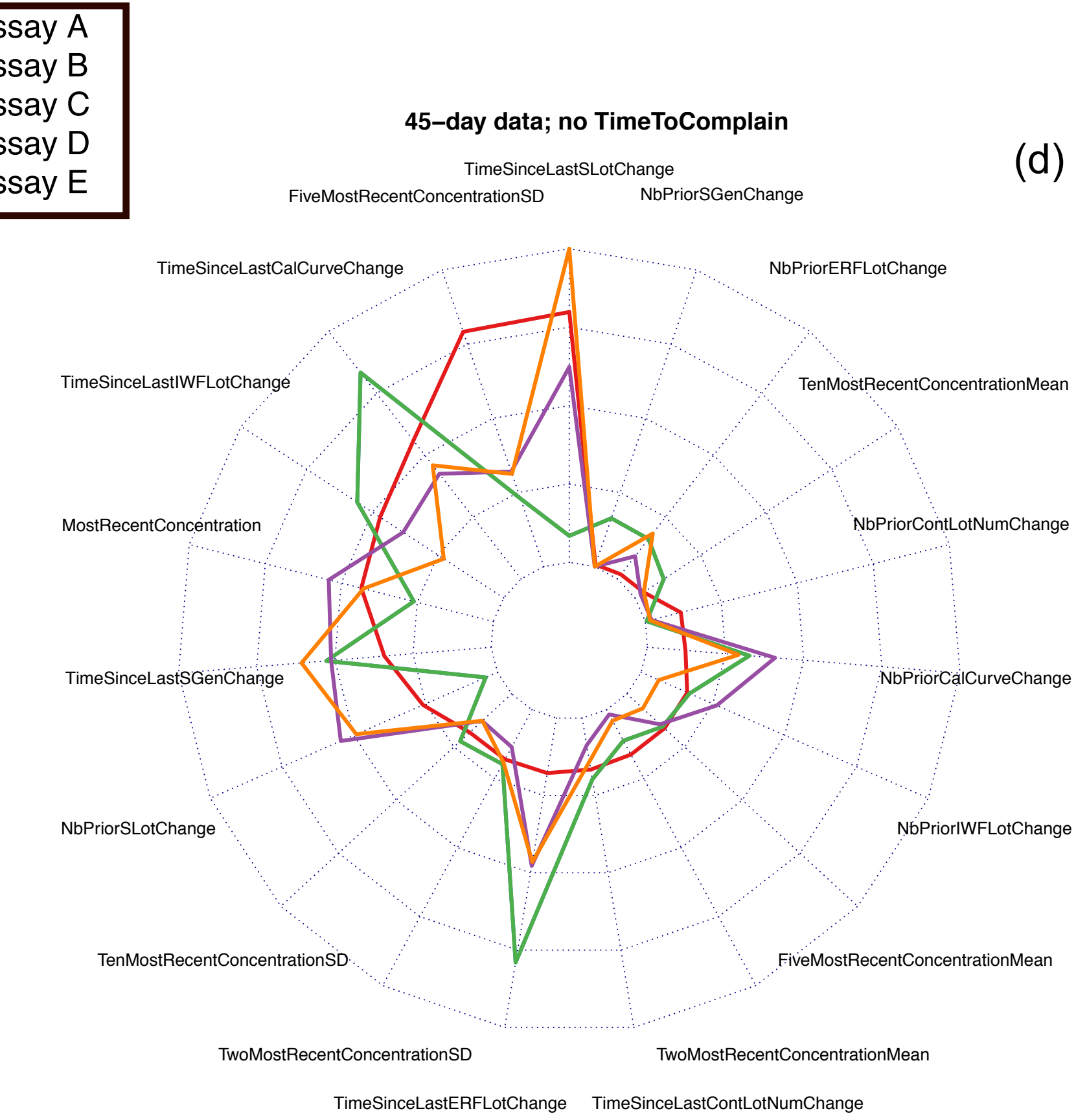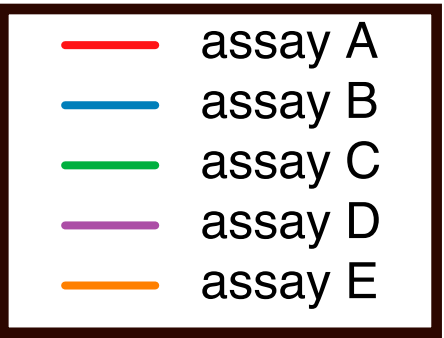

Supplement: Multimedia Appendix 9 [file medinform_v6i2e34_app9.pdf]
