# Supplementary material for: Predicting the Reasons of Customer Complaints: A First Step Toward Anticipating Quality Issues of In Vitro Diagnostics Assays with Machine Learning
Source: JMIR Med Inform. 2018 May 15;6(2):e34. doi: 10.2196/medinform.9960 (PMC5974458; doi:10.2196/medinform.9960)
Supplement: Multimedia Appendix 10 [file medinform_v6i2e34_app10.pdf]

assay B:	Observed Class											
Predicted Class	ACCH	ACCL	CORR	LINEAR	OK	OTHER	OUTLIERH	OUTLIERL	PREC	PROF	QCH	QCL
ACCH	95	8	4	3	24	1	5	2	8	0	31	18
ACCL	0	4	3	0	0	0	0	0	0	0	0	0
LINEAR	0	0	0	1	0	0	0	0	0	0	1	0
OK	18	1	2	1	124	0	3	3	3	0	5	6
OUTLIERH	0	0	0	0	2	0	9	0	0	0	0	0
QCH	1	0	0	0	2	0	0	1	0	0	25	1
QCL	0	1	0	0	0	0	0	0	0	0	0	3

assay E:	Observed Class										
Predicted Class	ACCH	ACCL	CAL	CORR	LEAKSPIL	OK	OTHER	OUTLIERH	PROF	QCH	QCL
ACCH	4	0	0	0	0	0	0	0	0	0	0
CAL	1	0	17	0	0	0	0	0	0	3	1
CORR	0	0	0	1	0	0	0	1	0	0	0
LEAKSPIL	0	0	0	0	5	0	0	0	0	0	0
OK	1	0	2	0	0	43	0	0	0	3	0
PROF	0	0	1	0	0	0	0	0	1	4	0
QCH	3	0	3	0	2	7	0	0	5	75	2
QCL	0	0	0	0	0	0	0	0	1	2	12

Multimedia Appendix 11. Examples of confusion tables obtained during cross-validation on the 90-day data, filtered for quality control (QC)only call areas (data not binned by QC level). Numbers on the diagonal show accurate predictions; false predictions are below the diagonal, whereas missed predictions are above.

Notes—ACCH: accuracy high, ACCL: accuracy low, CAL: calibration, CORR: CORRELATION, LEAKSPIL: LEAKAGE/SPILLAGE, OK: no issue, OTHER: other, OUTLIERH: outlier high, OUTLIERL: outlier low, PREC: precision, PROF: , QCH: QC high, QCL: QC low.
